# Parthenolide Is Neuroprotective in Rat Experimental Stroke Model: Downregulating NF-**κ**B, Phospho-p38MAPK, and Caspase-1 and Ameliorating BBB Permeability

**DOI:** 10.1155/2013/370804

**Published:** 2013-07-11

**Authors:** Lipeng Dong, Huimin Qiao, Xiangjian Zhang, Xiaolin Zhang, Chaohui Wang, Lina Wang, Lili Cui, Jingru Zhao, Yinxue Xing, Yanhua Li, Zongjie Liu, Chunhua Zhu

**Affiliations:** ^1^Department of Neurology, Second Hospital of Hebei Medical University, Shijiazhuang, Hebei 050000, China; ^2^Hebei Institute of Cardiocerebral Vascular Diseases, Shijiazhuang, Hebei 050000, China; ^3^Hebei Key Laboratory for Neurology, Shijiazhuang, Hebei 050000, China

## Abstract

Inflammatory damage plays an important role in cerebral ischemic pathogenesis and may represent a target for treatment. Parthenolide (PN) has been proved to elicit a wide range of biological activities through its anti-inflammatory action in the treatment of migraine, arthritis, and atherosclerosis. To decide whether this effect applies to ischemic injury in brain, we therefore investigate the potential neuroprotective role of PN and the underlying mechanisms. Male Sprague-Dawley rats were randomly divided into Saline, Vehicle, and PN groups and a permanent middle cerebral artery occlusion (MCAO) model was used. PN administered intraperitoneally immediately after cerebral ischemia and once daily on the following days. At time points after MCAO, neurological deficit, infarct volume, and brain water content were measured. Immunohistochemistry, western blot and RT-PCR were used to analyze the expression of NF-**κ**B and caspase-1 in ischemic brain tissue. Phospho-p38MAPK and claudin-5 were detected by western blot. The results indicated that PN dramatically ameliorated neurological deficit, brain water content, and infarct volume, downregulated NF-**κ**B, phospho-p38MAPK, and caspase-1 expressions, and upregulated claudin-5 expression in ischemic brain tissue. *Conclusions.* PN protected the brain from damage caused by MCAO; this effect may be through downregulating NF-**κ**B, phosho-p38MAPK, and caspase-1 expressions and ameliorating BBB permeability.

## 1. Introduction

Stroke is the third leading cause of death and the most frequent cause of permanent disability in adults worldwide [1]. The mechanisms that trigger ischemic brain damage could be related to excitotoxicity, oxidative stress, inflammation, apoptosis, and so on. The significance of the inflammatory response has been revealed during ischemic pathology in both animal and human stroke [2].

The mitogen-activated protein kinases (MAPKs), involving p38MAPK, and the transcription factor nuclear factor nuclear factor-kappa B (NF-*κ*B) contribute to the activation some cytokines and inducion of inflammation [3, 4]. Recently, we have proved that the activations of NF-*κ*B and p38MAPK are linked to the pathogenesis of cerebral ischemia [5–7]. The activations of NF-*κ*B and p38MAPK can be induced by caspase-1 independently of its enzymatic activity [8], which plays a key role in inflammatory pathways by processing prointerleukin-1*β* (IL-1*β*) into the active cytokine mature IL-1 [9]. After ischemic damage, the expression of caspase-1 is upregulated [10], leading to the generation of the active protein by oligomerization of procaspase-1 molecules and autoproteolytic cleavage into the active subunits [9, 11]. Previous researches using caspase-1-deficient transgenic mice have provided evidence for the role of caspase-1 in ischemic brain damage in vivo [12]. Inhibition of active caspase-1 with drugs can reduce brain damage and provide neuroprotective effects [13, 14].

**Figure 1:**
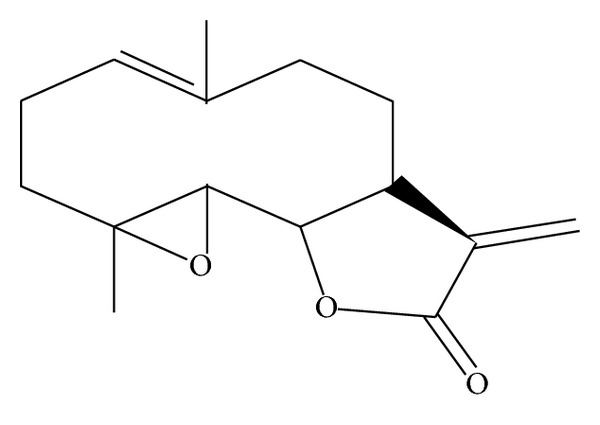
The chemical structure of PN.

Despite considerable advances in the understanding of the pathophysiology of cerebral vascular ischemia, therapeutic options for acute stroke are still limited. To find natural products, which can inhibit the inflammatory signal, may be a critical strategy for stroke therapy. Parthenolide (PN), the major *sesquiterpene lactone* found in Mexican Indian medicinal plants and in feverfew (*Tanacetum parthenium*), has demonstrated a wide range of pharmacological and biological activities, including anti-inflammation, antiapoptosis, and anti-oxidation. Importantly, its lipophilic character favored a good blood-brain barrier (BBB) permeability [15]. The structure of PN is clear as shown in Figure 1. PN has been used extensively for treatment of multiple inflammatory diseases, including fever and migraine with few mild side effects [16, 17]. Recent studies suggested that PN also exerted a protective effect on ischemia/reperfusion damage in heart [18]. However, the underlying mechanism of the protection of PN in cerebral ischemia remains unclear. In addition, the relationship between PN and caspase-1 and p38MAPK expressions in cerebral ischemia has not been investigated so far. We therefore investigated the potential protective effects of PN and the underlying mechanisms in ischemic stroke.

## 2. Materials and Methods

### 2.1. Animals and Ischemia Protocol

Male Sprague-Dawley rats (250–280 g) were supplied by the Laboratory Animal Centre of Hebei Medical University and were maintained on a 12 h light/12 h dark regime, with humidity of 60% ± 5%, 22 ± 3°C. All rats were provided with food and water ad libitum. The animals were acclimatized to the laboratory conditions for a period of at least 3 days before surgery. Animals were anesthetized by intraperitoneal injection of chloral hydrate (10%). Body temperature was monitored and maintained at 36.5°C to 37.5°C. A standard model of intraluminal middle cerebral artery (MCA) was used to make permanent (MCAO) focal ischemia by intraluminal placement of a filament as described previously [19, 20]. Sham-operated control rats received the same procedure except for filament insertion.

### 2.2. Experimental Groups and Drugs

All the rats were randomly divided into 6 groups: Saline groups: rats received sham operation or MCAO and equal volume of normal sodium; Vehicle groups: rats received sham operation (Sham) or MCAO (MCAO) and equal volume of equal volume of 0.05% Tween-80; PN groups: rats treated with PN (purity 94%, Sigma-Aldrich, USA) at 250 *μ*g/kg (Low) or 500 *μ*g/kg (High) intraperitoneally immediately after cerebral ischemia and then once daily on the following days. In preliminary experiments, neurological deficit, infarct volume, and brain water content were measured between Saline and Vehicle groups to exclude any biological effect of the vehicle. Because neurological deficit, infarct volume, and brain water content were comparable among Saline and Vehicle groups (data not shown), only Vehicle group was used for another experiment.

### 2.3. Neurological Deficit Score

A neurological test was administered by the same examiner blinded to the experimental groups at 24 h and 72 h (rats for determination of infarct volume and brain water content were included *n* = 10 per group per time point) after MCAO following a modified scoring system that was developed by Longa et al. as follows: 0, no deficits; 1, difficulty in fully extending the contralateral forelimb; 2, unable to extend the contralateral forelimb; 3, mild circling to the contralateral side; 4, severe circling; 5, falling to the contralateral side. The higher the neurological deficit score, the more the severe impairment of motor motion injury.

### 2.4. Brain Water Content

Brain water content was measured using the standard wet-dry method [21]. Rats were anesthetized with chloral hydrate (10%) (*n* = 6 per group per time point) and killed by decapitation at 24 h and 72 h after MCAO. The brains were quickly removed and placed on a dry surface. After dissecting free 4 mm thickness of frontal pole, a coronal brain slice was cut by brain slicer (Beijing Sunny Instruments Co., Ltd., Beijing, China) with 3 mm thick and the slice was divided into the ipsilateral and contralateral hemispheres. The two hemisphere slices packaged with preweighed tin foil were immediately weighed on an electronic balance to obtain the wet weight, dried for 24 h in an oven at 100°C, and then reweighed to obtain the dry weight. Brain water content was calculated as a percentage using the following formula: (wet weight − dry weight)/wet weight × 100%.

### 2.5. Brain Infarct Volume

Infarct volume after MCAO was determined by 2,3,5-triphenyltetrazolium chloride (TTC) at 24 h and 72 h after MCAO (*n* = 6 per group per time point). Animals were euthanized and the brains were quickly collected. Brain tissue was sliced into five coronal sections (3 mm thick) and stained with a 2% solution of TTC at 37°C for 20 min [22], followed by fixation with 4% paraformaldehyde. Normal tissue was stained deep red, while the infarct area was stained a pale gray color. TTC-stained sections were photographed and the digital images were analyzed using image analysis software (Image-Pro Plus 5.1; Media Cybernetics, Inc., Bethesda, MD, USA) to calculate the infarct volume. To compensate for the effect of brain edema, the percentage hemisphere lesion volume was calculated by the following formula [23]: %HLV = {[total infarct volume − (volume of intact ipsilateral hemisphere − volume of intact contralateral hemisphere)]/contralateral hemisphere volume} × 100%. 

### 2.6. Immunohistochemical Staining (IHC)

Paraffin-embedded sections were used to assess the expression of caspase-1, phospho-p38MAPK, and NF-*κ*B p65, according to the standard histological procedures described previously [24, 25] at 24 h and 72 h after MCAO (*n* = 6 per group per time point). Briefly, brains were fixed in 4% paraformaldehyde in phosphate-buffered saline (PBS; 0.01 M, pH 7.4) over 24 h at 4°C and then dehydrated in a graded series of alcohols and embedded in paraffin. Brain tissues were cut at 5 *μ*m using a Leica RM1850 Rotary Microtome (Leica Microsystem, Hesja, Germany). Brain sections were incubated in 3% H_2_O_2_ to eliminate endogenous peroxidase activity and 3% normal goat serum and then incubated with caspase-1 mouse monoclonal antibody (1 : 100, Santa Cruz Biotechnology), phospho-p38MAPK (Tyr180/Tyr182) (1 : 100, Cell Signaling Technology) and NF-*κ*B p65 rabbit polyclonal antibody (1 : 150, Santa Cruz Biotechnology) in 0.01 mol/L PBS overnight at 4°C. They were rinsed with PBS and incubated with secondary antibodies at 37°C for 45 min. They were rinsed again with PBS and incubated with secondary biotinylated conjugates at 37°C. Slices were developed with diaminobenzidine and counterstained with hematoxylin. The secondary antibodies, secondary biotinylated conjugates, and diaminobenzidine from the SP kit (Zhongshan Biology Technology Company, Beijing, China) were used to visualize the signals. The immunoreactive cells were counted under a 400x light microscope in five visual fields of the ischemic cortex region around the infarct core. The average number was used for statistical analysis and represented the immunopositive cells of that rat (*n* = 6 per group per time point).

### 2.7. Western Blot

Protein extraction was obtained using a Total Protein Extraction Kit and Nuclear-Cytosol Extraction Kit (Applygen Technologies Inc., Beijing, China) following the manufacturers' protocols at 24 h and 72 h after MCAO (*n* = 6 per group per time point), as described previously by our laboratory [25]. Total protein for active caspase-1, p38MAPK, and phospho-p38MAPK and nuclear protein for NF-*κ*B were prepared. Protein concentration of the supernatant was determined using a BCA Protein Assay Reagent Kit (Novagen, Madison, WI, USA) with bovine serum albumin as the standard. An equivalent amount of 50 *μ*g total protein samples, as well as 30 *μ*g nuclear protein samples, was subjected to electrophoresis on 10% sodium dodecyl sulfate-polyacrylamide gels (SDS/PAGE) for 45 min at 80 V followed by 100 min at 100 V and then transferred onto PVDF membranes (Millipore Corporation, USA) for 2 h at 100 V. The membranes were blocked with 5% skimmed milk/TPBS (10 mM Tris-HCl, 150 mM NaCl, 0.05% Tween-20) for 2 h at room temperature, then incubated overnight at 4°C with anti-caspase-1 (1 : 100, Santa Cruz Biotechnology), anti-phosphorylated p38MAPK (1 : 500, Cell Signaling Technology), anti-p38MAPK (1 : 500, Cell Signaling Technology), anti-NF-*κ*B P65 antibody (1 : 500, Santa Cruz Biotechnology), and anti-claudin-5 antibody (1 : 200, Santa Cruz Biotechnology, CA, USA) diluted in 1% BSA/TPBS. Polyclonal rabbit anti-beta actin antibody (1 : 500, Zhongshan Biotechnology) was used as an internal control. In the following day, membranes were washed with PBS containing 0.1% Tween-20 (TPBS) (10 min ×3) each time and subsequently incubated in TPBS containing fluorescent labeling second antibodies (IRDye 800-conjugate rabbit anti-goat, goat anti-rabbit IgG or anti-mouse 1 : 5000 dilution; Rockland, Gilbertsville, PA, USA) for 1 h at room temperature. Membranes were then washed three times with TPBS (10 min ×3) and the relative density of bands was analyzed on an Odyssey infrared scanner (LICOR Bioscience, Lincoln, NE, USA). Densitometric values were normalized with respect to beta actin immunoreactivity to correct any loading and transfer differences among samples.

### 2.8. Reverse Transcription-Polymerase Chain Reaction (RT-PCR)

Total RNA from cortex supplied by right MCA was extracted using Trizol reagent (Invitrogen, Carlsbad, CA, USA) following the manufacturer's instructions. RNA (2 *μ*g) of each sample was reverse transcribed for synthesizing cDNA. The cDNA (1 *μ*L) was then amplified by PCR. Primers were synthesized by Shanghai Sangon Biological Engineering Technology Company Limited (Table 1). Reverse transcription used reagents from Promega following the manufacturer's instructions. PCR conditions were initial denaturation for 2 min at 95°C, 35 cycles of amplification with denaturation at 95°C for 30 seconds, annealing at 52°C for 30 seconds, and extension at 72°C for 40 seconds, followed by a final step of 5 min at 72°C. RT-PCR products (5 *μ*L) were separated on 2% agarose gel. The gray scale of the electrophoresis strip was scanned by an ultraviolet photometry (UVP) gel imaging system. The intensity of each band was quantified using SynGene software, and the ratios of each gene product normalized to *β*-actin product were considered as the expression of each gene (*n* = 6 for each group per time point).

### 2.9. Statistical Analysis

All data were analyzed using SPSS 13.0 software. Quantitative data were expressed as mean ± SD. Statistical comparisons were performed by one-way ANOVA followed by Student-Newman-Keuls and LSD tests for multiple comparisons. For neurological deficit, Mann-Whitney *U* test was used for comparisons between two groups. Differences with *P* < 0.05 were considered statistically significant.

## 3. Results

### 3.1. PN Improved Neurological Deficit

**Figure 2:**
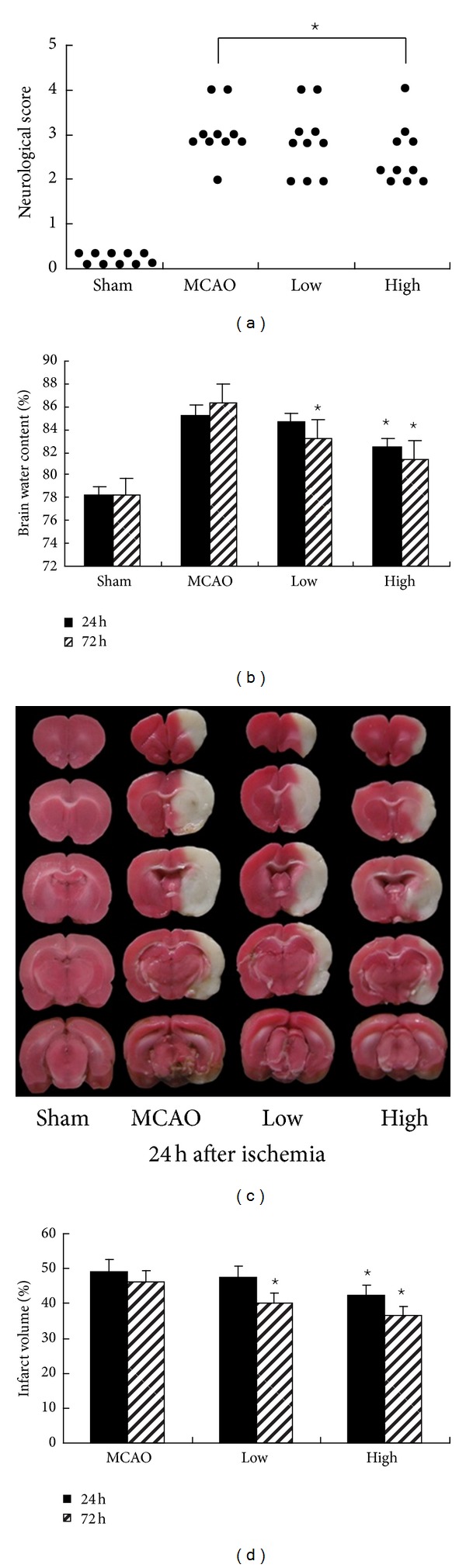
PN improved neurological outcome after MCAO. (a) shows the neurological deficit scores at 24 h. Each circle represented the score for a single rat. Compared with MCAO group, the neurological deficit scores were significantly decreased in High group at 24 h and 72 h. ^⋆^ *P* < 0.05  versus MCAO group. (b) shows the water content of ipsilateral hemispheres. Compared with MCAO group, the brain water content of ipsilateral hemispheres significantly was reduced in High group at 24 h and 72 h, but only at 72 h in Low group after MCAO ^⋆^ *P* < 0.05   versus MCAO group. (d) shows infarct volume at 24 h and 72 h. Compared with MCAO group, the infarct area was significantly reduced in High group at 24 h and 72 h, but only at 72 h in Low group after MCAO. ^⋆^ *P* < 0.05  versus MCAO group. Representative photographs in (c) shows brain slices stained with TTC.

Neurological deficit was examined and scored on a 6-point scale at 24 h and 72 h after MCAO and Mann-Whitney *U* test analysis was conducted (Figure 2(a)). Following MCAO, there was a significant improvement in neurological function scores in the high dose group (High) compared with the MCAO group (*P* < 0.05). By contrast, the scores in low dose group (Low) were not lowered by PN (*P* > 0.05).

### 3.2. PN Reduced Brain Edema

Wet-dry method was used to measure brain water content. Brain edema in the ischemic hemisphere of each treatment group is shown in Figure 2(b). There was a significant increase in brain water content in the MCAO group compared with the Sham group at all time points (*P* < 0.05 for all). The two doses of PN decreased the percentage of brain water content in ipsilateral hemispheres after stroke. Compared with MCAO group, high dose of PN reduced the brain water content of ipsilateral hemispheres significantly (24 h: 82.44% ± 0.48% versus 85.25% ± 0.50%, 72 h: 81.34% ± 0.51% versus 86.24% ± 0.38%, *P* < 0.05, Figure 2(b)). In Low group, the brain water content was reduced (Low versus MCAO: 83.24% ± 0.34% versus 86.24% ± 0.38%, *P* < 0.05, Figure 2(b)) at 72 h. 

### 3.3. PN Reduced Infarct Volume

The neuroprotective effects of PN were also evaluated by measuring infarct volumes at 24 h and 72 h after ischemia (Figures 2(c) and 2(d)). No infarction was observed in Sham group. Extensive lesion was found in both striatum and cortex in MCAO group (*P* < 0.05 versus Sham group). In High group, infarct size was significantly reduced (24 h: 42.35% ± 1.34% versus 49.15% ± 0.78%, 72 h: 36.67% ± 0.71% versus 46.32% ± 0.38%, *P* < 0.05 for all, Figure 2(d)). In Low group, the brain infarct size was reduced (Low versus MCAO: 40.20% ± 0.45% versus 46.32% ± 0.38%, *P* < 0.05, Figure 2(d)) at 72 h.

### 3.4. PN Inhibited Ischemia-Induced Caspase-1, Phospho-p38MAPK, and NF-*κ*B Activation

**Figure 3:**
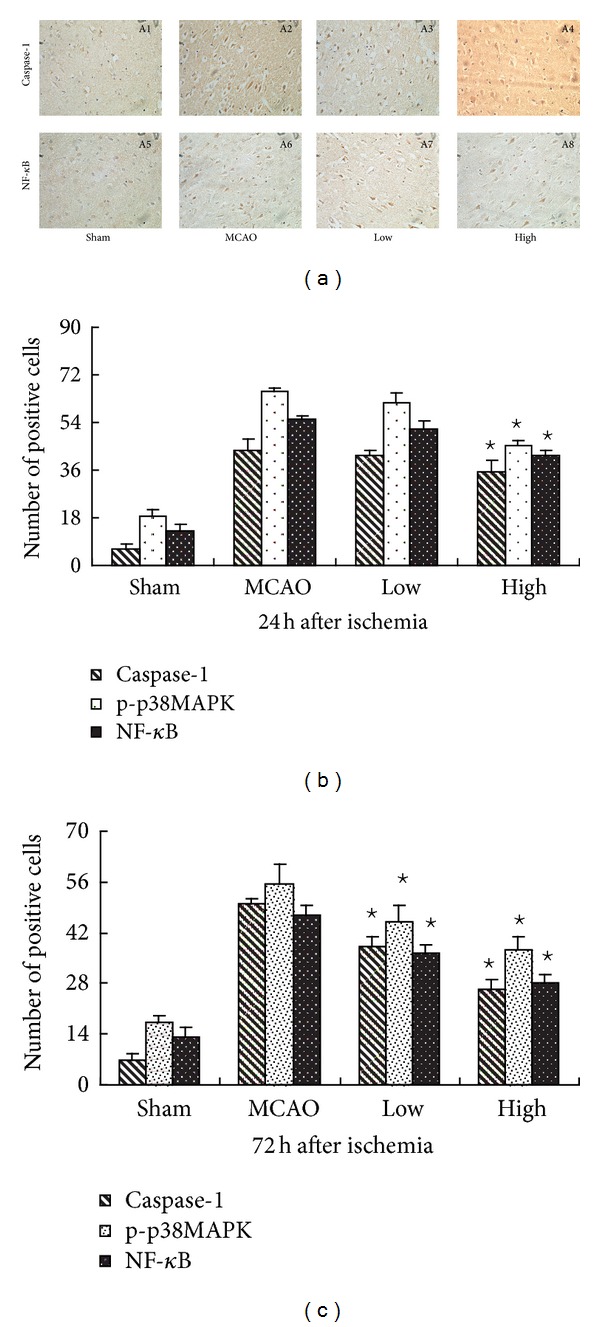
PN decreased the positive cells of caspase-1 and NF-*κ*B after MCAO. (b) and (c) shows the positive cells of caspase-1, phospho-p38MAPK, and NF-*κ*B. Compared with MCAO group, the number of positive cells of caspase-1, phospho-p38MAPK, and NF-*κ*B was significantly decreased in High group at 24 h and 72 h, but only at 72 h in Low group after MCAO ^⋆^ *P* < 0.05  versus MCAO group. Representative photographs in (a) show the expression of caspase-1 (A1, A2, A3, A4) and NF-*κ*B (A5, A6, A7, A8) at 72 h after ischemia (400x).

**Figure 4:**
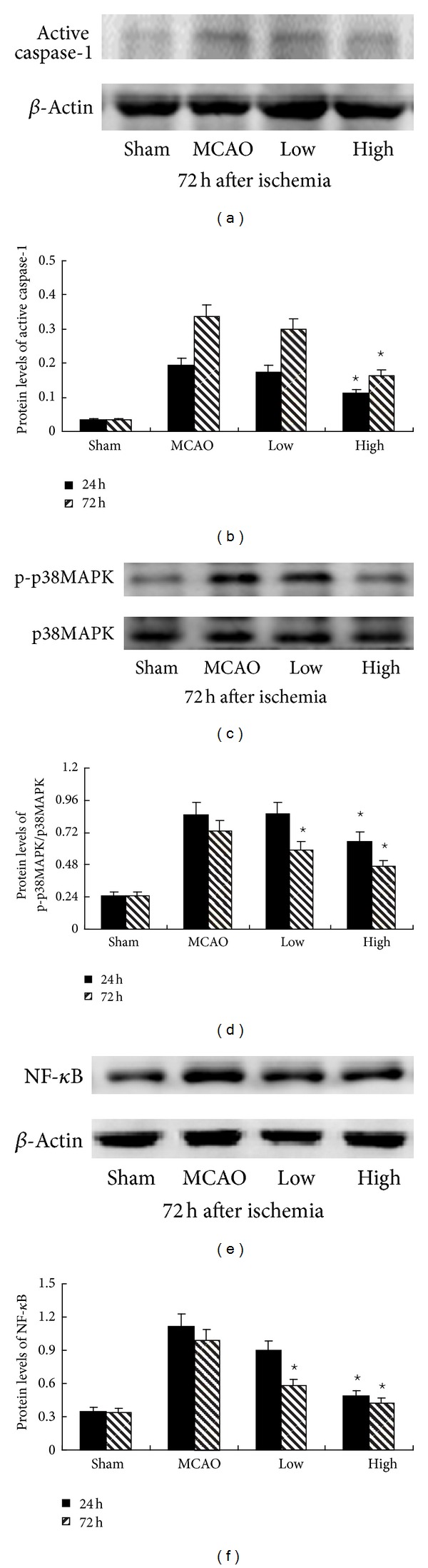
PN decreased protein of active caspase-1, phospho-p38MAPK, and nuclear NF-*κ*B p65 after MCAO. (b) shows the expression of active caspase-1. Compared with MCAO group, the expression of active caspase-1 was significantly decreased in High group at 24 h and 72 h. (d) shows the expression of phospho-p38MAPK. (f) shows the expression of nuclear protein NF-*κ*B. Compared with MCAO group, the expression of phospho-p38MAPK and nuclear protein NF-*κ*B was significantly decreased in High group both at 24 h and 72 h, but decreased only at 72 h in Low group ^⋆^ *P* < 0.05 versus MCAO group. Representative photographs in ((a), (c), (e)) show the influence of PN on protein of active caspase-1, phospho-p38MAPK, and nuclear protein NF-*κ*B at 72 h.

**Figure 5:**
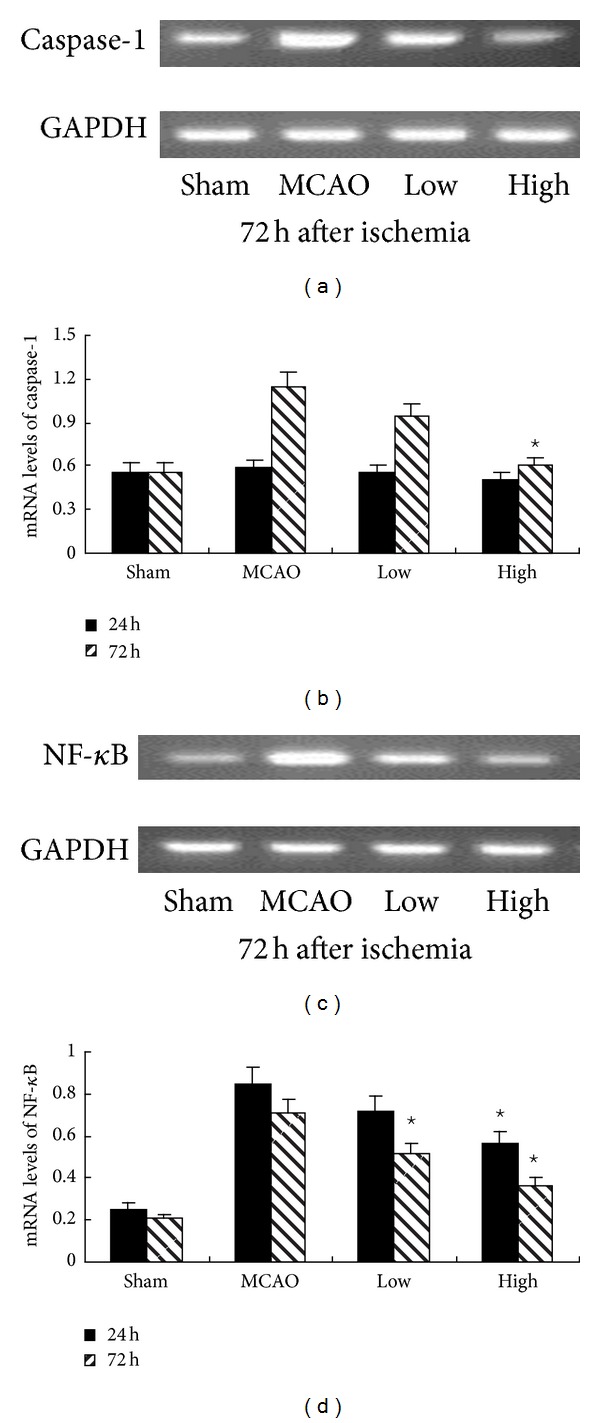
PN decreased mRNA expressions of caspase-1 and NF-*κ*B after MCAO. (b) shows the mRNA expression of caspase-1. Compared with MCAO group, the expression of caspase-1 was significantly decreased in High group 72 h. There is no difference between sham and MCAO group at 24 h. (d) shows the mRNA expression of NF-*κ*B. Compared with MCAO group, the expression of NF-*κ*B was significantly decreased in High group both at 24 h and 72 h, but decreased only at 72 h in Low group ^⋆^ *P* < 0.05 versus MCAO group. Representative photographs in ((a) and (c)) show the influence of PN on the mRNA expression of NF-*κ*B and caspase-1 at 72 h.

Immunohistochemistry showed the expression of caspase-1 and NF-*κ*B at 72 h after ischemia in Figure 3(a). As expected, the numbers of positive cells of caspase-1, phospho-p38MAPK, and NF-*κ*B were significantly decreased compared with MCAO group in High group at 24 h and 72 h, but only at 72 h in Low group after MCAO (*P* < 0.05, Figure 3). In agreement with the results of immunohistochemistry, western blot also showed a significant decrease of nuclear protein NF-*κ*B and phospho-p38MAPK in High group at 24 h and 72 h, but only at 72 h in Low group after MCAO (*P* < 0.05, Figures 4(c)–4(f)). Western blot for active caspase-1 (p20) showed that PN reduced the levels of active caspase-1 (p20) in High group (*P* < 0.05, Figures 4(a) and 4(b)), but there is no significant difference in Low group at 24 h and 72 h (*P* < 0.05, Figures 4(a) and 4(b)). RT-PCR showed that the expression of caspase-1 was decreased in the presence of PN at 72 h (*P* < 0.05, Figure 5). There was no difference between Sham group and MCAO group at 24 h (*P* > 0.05, Figure 5(b)). Following western blot, RT-PCR showed the same results on the expression of NF-*κ*B (Figures 5(c) and 5(d)).

### 3.5. PN Promoted the Expression of Claudin-5

**Figure 6:**
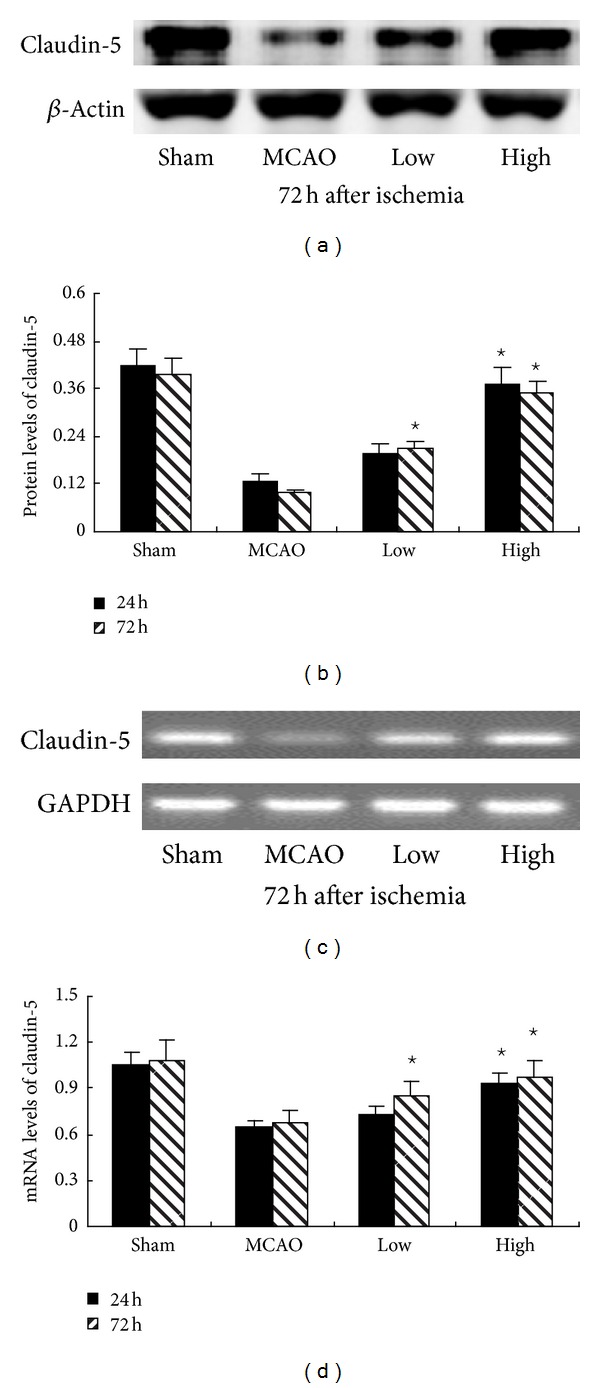
PN increased the expression of claudin-5 after MCAO. The expression of claudin-5 was assessed by western blot ((a) and (b)) and reverse transcription-polymerase chain reaction ((c) and (d)). Compared with MCAO group, the expression of claudin-5 was significantly increased in High group both at 24 h and 72 h, but increased only at 72 h in Low group ^⋆^ *P* < 0.05 versus MCAO group.

To examine whether PN ameliorated BBB permeability, the expressions of claudin-5 were detected by western blot and RT-PCR at both protein and mRNA levels. As Figure 6 showed, the expression of claudin-5 in cerebral ischemia was significantly upregulated at both protein and mRNA levels after treatment with high dose of PN at 24 h and 72 h (*P* < 0.05). However, the upregulation of claudin-5 was only observed in Low group at 72 h (*P* < 0.05).

## 4. Discussion

The experimental results proved that PN might protect against ischemic brain damage by improving neurological dysfunction, reducing infarct size, edema, and pathological changes after MCAO; the underlying mechanism of this neuroprotection might be involved inhibition of caspase-1, phospho-p38MAPK, and NF-*κ*B expressions; PN might ameliorate BBB permeability.

It is well known that MCAO is a classical model of cerebral ischemia [20, 26]. Inflammatory response significantly contributes to ischemic brain damage, occurring within minutes after onset of cerebral ischemia [27, 28]. Increasing evidence implicates that inflammation causes secondary ischemic brain damage [29], with a further worsening of edema. For example, in our recent studies it was demonstrated that the systemic administration of curcumin, oxymatrine, and luteolin during cerebral infarction, which have been proved to be anti-inflammatory agents, could improve neurological deficit, alleviate brain edema and infarct sizes, and regulate cytokines expression in cortex, such as downregulating p38MAPK, NF-*κ*B, Toll-like receptor-2,4,5, and myeloid differentiation factor 88 [7, 30, 31]. This study showed that PN reduced the infarct size and edema and suppressed inflammatory response after ischemic stroke, which is in agreement with previous reports about the effects of PN on ischemic cardiac damage in rats [18].

It has been proved that PN can inhibit proinflammatory cytokines, such as TNF-*α*, IL-1, COX-2, and MCP-1 in vitro [32, 33]. Consistent with the idea that PN inhibits progression of the inflammatory response, it reduces cardiovascular damage in endotoxic shock, retards atherosclerotic lesions, and has beneficial effects in myocardial ischemia in vivo [18, 33, 34]. It has been shown that PN is a direct inhibitor of the protease activity of caspase-1 by alkylating critical cysteine residues in the p20 subunit [35]. In addition, it is a good inhibitor of the Nlrp3 inflammasome and that this activity is independent of the inhibitory effect of PN on the NF-*κ*B pathway. Pretreatment with PN (1 mg/kg) 1 h before the LPS-challenge can reduce brain inflammatory response [36]. However, any effect of PN was not found on LPS-induced fever, when injecting it (2 mg/kg) 30 min before injection of LPS that was used in mice [37]. In the present study, we found that high dose of PN (500 *μ*g/kg) had a significant effect on the brain inflammation after ischemic stroke. Low dose of PN also exerted some moderately beneficial effects in stroke-related rat brain damage. In summary, the effect of PN in permanent cerebral ischemia will be well explained in larger number of animals or possibly in bigger animals as well as humans.

A growing body of evidence suggests that NF-*κ*B, activating p38MAPK, is an important transcription factor responsible for immune and inflammatory response [4]. Several studies have suggested that NF-*κ*B plays a pivotal role, responsible in part for “turning on” genes, such as IL-1, TNF-*α*, and MCP-1 [3]. NF-*κ*B is upregulated shortly which contributes to cerebral injury induced by ischemia [7, 25, 31]. Inhibition of NF-*κ*B activation attenuates inflammatory response [38]. Some reports report that intraventricular administration of the inhibitor of p38MAPK (SB203580) can result in a reduction in BBB disruption, edema formation, and infarct volume [39]. Our study showed that PN could suppress the activity of phospho-p38MAPK and NF-*κ*B. 

Caspases mediate cell death and inflammation as intracellular cysteine proteases. Activation of one protease can result in cleavage and activation of additional molecules of the same or other proteases, with an amplified protease cascade. Our work focused on caspase-1, because its “apical” position within the enzymatic cascade of caspases makes it potentially important therapeutic target. Oligomerization of procaspase-1 molecules includes the formation of a macromolecular complex known as the inflammasome [40, 41]. Blocking the formation of the inflammasome complex is a novel upstream anti-inflammatory therapeutic strategy that involves inhibition of caspase-1 activation [42]. Taken together, blockade of caspase-1 may represent an intriguing way to alleviate inflammatory responses to focal ischemia. In this study, it was found that NF-*κ*B, phospho-p38MAPK, and caspase-1 activation in the cortex were activated after MCAO, with agreement to previous results [5, 6, 43]. As expected, inhibiting NF-*κ*B and phospho-p38MAPK activation, PN also inhibited the expression of caspase-1, leading to downregulating inflammation, with delaying lesion growth in the ischemic area. This study indicated that the protective effect of PN against cerebral ischemic injury, contributed, at least in part, to the lessening of ischemia-induced proinflammatory factors. In the light of inhibiting caspase-1, whether the inflammasome pathway as a novel upstream pathway is also inhibited by PN needs further research in vivo.

BBB is a dynamic interface between the peripheral circulation and the central nervous system (CNS). Tight junctions (TJs) are important structural components of the BBB maintained by transmembrane TJ proteins, such as occludin and claudins [44]. Disruption of BBB after cerebral ischemia is considered to be the initial step in the development of brain injuries. An increase in the tight junctional protein claudins or a decrease in the tyrosine phosphorylation of occludin has been shown to cause a decrease in BBB leakage [45, 46]. Claudin-5 is a capital cell adhesion molecule of TJs in brain endothelial cells. In view of the basal role of claudin-5 in regulating BBB permeability and sustaining integrity of cerebrovascular endothelial cells, we estimated whether PN exerted an impact on claudin-5. We found that PN upregulated the expression of claudin-5 at protein and mRNA levels. It suggests that PN may have a potential in attenuating the disruption of BBB after ischemia. However, the involved mechanism was unclear and needed to be further studied.

## 5. Conclusion

Systemic administration of PN alleviates ischemic brain injury by inhibiting inflammatory response and this may occur through downregulating the expressions of caspase-1, phospho-p38MAPK, and NF-*κ*B and ameliorating BBB through upregulation of claudin-5.

## Figures and Tables

**Table 1:** Summary of the RT-PCR primers sequences.

Gene	Primers	Sequences
Caspase-1	Forward	5′-ACACGTCTTGCCCTCATTATCT-3′
	Reverse	5′-ATAACCTTGGGCTTGTCTTTCA-3′
NF-*κ*B	Forward	5′-CGATCTGTTTCCCCTCATCT-3′
	Reverse	5′-ATTGGGTGCGTCTTAGTGGT-3′
Claudin-5	Forward	5′-CACAGAGAGGGGTCGTTGAT-3′
	Reverse	5′-ACTGTTAGCGGCAGTTTGGT-3′
GAPDH	Forward	5′-GCCATGTACGTAGCCATCCA-3′
	Reverse	5′-GAACCGCTCATTGCCGATAG-3′
